# Supplementary material for: Anti-poverty policy and health: Attributes and diffusion of state earned income tax credits across U.S. states from 1980 to 2020
Source: PLoS One. 2020 Nov 20;15(11):e0242514. doi: 10.1371/journal.pone.0242514 (PMC7678980; doi:10.1371/journal.pone.0242514)
Supplement: S1 Appendix — (PDF) [file pone.0242514.s001.pdf]

Codebook for Earned Income Tax Credit

Prepared by the Policy Surveillance Program Staff

July 2016

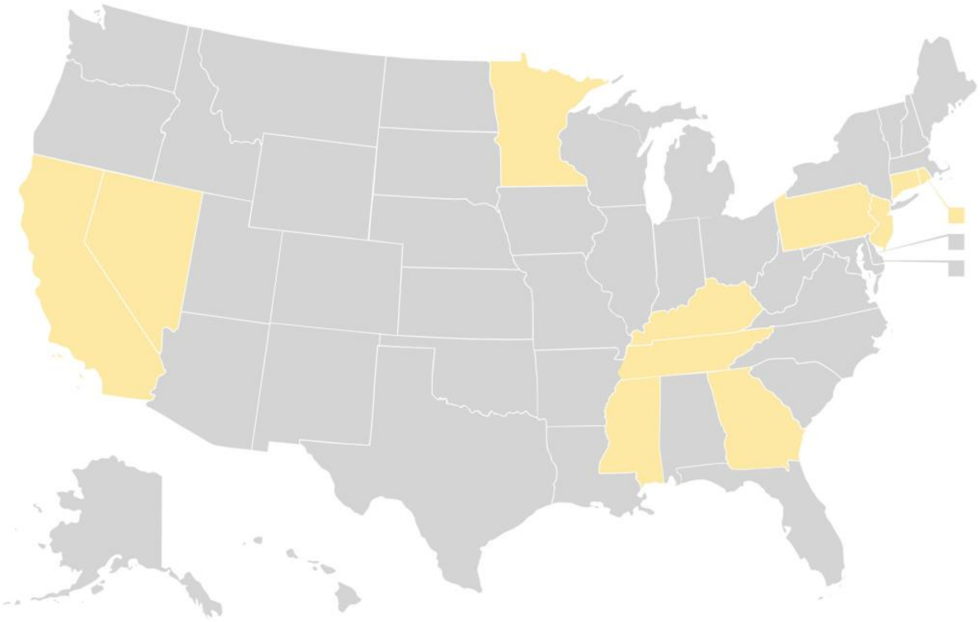

CODEBOOK
July 2016

Earned Income Tax Credit

This codebook describes the variables in a dataset that examines variation in state laws regulating involuntary outpatient commitment. This dataset is longitudinal and covers the period of time between January 1, 1980 and February 1, 2016. The jurisdictions selected for measurements are the 50 states and the District of Columbia.

There are six standard variables in every LawAtlas dataset. They are:

ID:
An identification code generated by the LawAtlas Workbench.

FIPS CODE:
The Federal Information Processing Standard (FIPS) codes are a standardized set of numeric codes issued by the National Institute of Standards and Technology to ensure uniform identification of geographic entities through all federal government agencies.

JURISDICTION:
This is a dropdown selection in each coding form. It includes the jurisdictions coded in the dataset.

EFFECTIVE DATE:
This date (MM/DD/YYYY) is the most recent effective date of the legal text captured for this place. The effective date represents the date the policy coded was put into effect.

VALID THROUGH DATE:
This date (MM/DD/YYYY) is the last date the policy was in effect as reflected by the legal text captured to code this policy.

LEGAL TEXT:
This is a text box that captures all legal text relevant to the coding questions. This will not download into Microsoft Excel.

Questions	
Question 1:	What is the Iteration?
Question Type:	Numeric field
Variable Name:	EITCIteration
Variable Values:	N/A
Question 2:	Does the jurisdiction have an EITC Law?
Question Type:	Binary - mutually exclusive
Variable Name:	EITC_Law
Variable Values:	0, 1
Value Label:	0 = No
Value Label:	1 = Yes
Question 3:	What law governs an individual's eligibility requirements for the EITC?
Question Type:	Categorical - mutually exclusive
Variable Name:	EITC_Eligble
Variable Values:	1, 2, 3
Value Label:	1 = Federal Law
Value Label:	2 = Both Federal & State Law
Value Label:	3 = State Law
Question 4:	What are the additional state requirements?
Question Type:	Categorical - check all that apply
Variable Name:	EITC_AddReq_Taxpayer cannot claim EITC if taxpayer claims another tax credit
Variable Values:	0, 1
Value Label:	0 = No
Value Label:	1 = Yes
Question 5:	What are the additional state requirements?
Question Type:	Categorical - check all that apply
Variable Name:	EITC_AddReq_State budget requirements must be met for payment
Variable Values:	0, 1
Value Label:	0 = No
Value Label:	1 = Yes
Question 6:	What are the additional state requirements?
Question Type:	Categorical - check all that apply
Variable Name:	EITC_AddReq_Married taxpayers must file jointly
Variable Values:	0, 1
Value Label:	0 = No
Value Label:	1 = Yes
Question 7:	What are the additional state requirements?

Question Type:	Categorical - check all that apply
Variable Name:	EITC_AddReq_Taxpayer must earn a minimum dollar amount of earned income
Variable Values:	0, 1
Value Label:	0 = No
Value Label:	1 = Yes
Question 8:	What are the additional state requirements?
Question Type:	Categorical - check all that apply
Variable Name:	EITC_AddReq_Taxpayer must claim federal credit
Variable Values:	0, 1
Value Label:	0 = No
Value Label:	1 = Yes
Question 9:	What are the additional state requirements?
Question Type:	Categorical - check all that apply
Variable Name:	EITC_AddReq_Taxpayer disqualified if required to pay alternative minimum tax
Variable Values:	0, 1
Value Label:	0 = No
Value Label:	1 = Yes
Question 10:	What are the additional state requirements?
Question Type:	Categorical - check all that apply
Variable Name:	EITC_AddReq_No additional state requirements explicitly stated in the law
Variable Values:	0, 1
Value Label:	0 = No
Value Label:	1 = Yes
Question 11:	What is the maximum investment income allowed to qualify for the EITC?
Question Type:	Categorical - mutually exclusive
Variable Name:	EITC_MaxInvest
Variable Values:	1, 2
Value Label:	1 = Adopts federal law
Value Label:	2 = The maximum investment income allowed is not specified in the law
Question 12:	What happens when the EITC exceeds a taxpayer's tax liability?
Question Type:	Categorical - mutually exclusive
Variable Name:	EITC_Refund
Variable Values:	1, 2, 3, 4, 5
Value Label:	1 = taxpayer receives full refund
Value Label:	2 = taxpayer receives partial refund
Value Label:	3 = the credit is not refundable, but can be applied against future tax liability

Value Label:	4 = the credit is not refundable and cannot be applied to future tax liability
Value Label:	5 = not specified in the law
Question 13:	Does the law provide an EITC for single individuals with no children?
Question Type:	Binary - mutually exclusive
Variable Name:	EITC_SingNoChild
Variable Values:	0, 1
Value Label:	0 = No
Value Label:	1 = Yes
Question 14:	What is the required age for single individuals with no children?
Question Type:	Categorical - mutually exclusive
Variable Name:	EITC_SingNoChildAge
Variable Values:	1, 2, 3
Value Label:	1 = Adopts federal law
Value Label:	2 = Specified in state law
Value Label:	3 = Required age not specified in the law
Question 15:	What is the minimum age?
Question Type:	Numeric field
Variable Name:	EITC_SingNoChildMinAge
Variable Values:	N/A
Question 16:	What is the maximum age?
Question Type:	Numeric field
Variable Name:	EITC_SingNoChildMaxAge
Variable Values:	N/A
Question 17:	How is the credit applied to tax liability?
Question Type:	Categorical - check all that apply
Variable Name:	EITC_CreditTypeSingNo_Dollar Amount
Variable Values:	0, 1
Value Label:	0 = No
Value Label:	1 = Yes
Question 18:	How is the credit applied to tax liability?
Question Type:	Categorical - check all that apply
Variable Name:	EITC_CreditTypeSingNo_Percentage
Variable Values:	0, 1
Value Label:	0 = No
Value Label:	1 = Yes
Question 19:	How is the credit applied to tax liability?

Question Type:	Categorical - check all that apply
Variable Name:	EITC_CreditTypeSingNo_Exempt from State Income Tax
Variable Values:	0, 1
Value Label:	0 = No
Value Label:	1 = Yes
Question 20:	How is the credit applied to tax liability?
Question Type:	Categorical - check all that apply
Variable Name:	EITC_CreditTypeSingNo_Credit type not specified in law
Variable Values:	0, 1
Value Label:	0 = No
Value Label:	1 = Yes
Question 21:	What is the dollar amount of the credit?
Question Type:	Currency Field
Variable Name:	EITC_SingNoChildDollAmt
Variable Values:	N/A
Question 22:	What is the EITC percentage for single individuals with no children?
Question Type:	Numeric Field
Variable Name:	EITC_SingNoChildPercent
Variable Values:	N/A
Question 23:	What is the EITC percentage applied to?
Question Type:	Categorical- Mutually Exclusive
Variable Name:	EITC_SingNoChildPercentApplied
Variable Values:	1, 2, 3, 4, 5, 6
Value Label:	1 = Federal EITC
Value Label:	2 = Total Earned Income
Value Label:	3 = EITC Credit Fund
Value Label:	4 = $12,000 minus individual total income
Value Label:	5 = State Income Tax Liability
Value Label:	6 = Not applicable
Question 24:	What is the EITC phase out percentage for single individuals with no children?
Question Type:	Numeric Field
Variable Name:	EITC_SingNoChildPhaseoutPercent
Variable Values:	N/A
Question 25:	What is the earned income amount?
Question Type:	Categorical - mutually exclusive
Variable Name:	EITC_SingNoChildAmount

Variable Values:	1, 2, 3, 4
Value Label:	1 = Adopts federal law
Value Label:	2 = Specified in state law
Value Label:	3 = Ten thousand dollars or less
Value Label:	4 = Earned income amount not specified in the law
Question 26:	What is the dollar amount of the credit?
Question Type:	Currency Field
Variable Name:	EITC_SingNoChildDollAmount
Variable Values:	N/A
Question 27:	What is threshold phase out AGI for single individuals with no children?
Question Type:	Categorical - mutually exclusive
Variable name:	eitc-singnochildthrphaseagi
Value Label:	1 = Adopts federal law
Value Label:	2 = Specified in state law
Value Label:	3 = Threshold phase out AGI not specified in the law
Question 28:	What is the applicable dollar amount?
Question Type:	Currency Field
Variable Name:	eitc-singnochildthrphaseagiamt
Variable Values:	N/A
Question 29:	What is the maximum EITC eligible AGI for single individuals with no children?
Question Type:	Categorical - mutually exclusive
Variable Name:	EITC_SingNoChildMaxAGI
Variable Values:	1, 2, 3
Value Label:	1 = Adopts federal law
Value Label:	2 = Specified in state law
Value Label:	3 = Maximum EITC eligible AGI not specified in the law
Question 30:	What is the applicable dollar amount?
Question Type:	Currency Field
Variable Name:	EITC_SingNoChildMaxAGIAmt
Variable Values:	N/A
Question 31:	Does the law provide an EITC for single individuals with one child?
Question Type:	Binary - mutually exclusive
Variable Name:	EITC_SingOneChild
Variable Values:	0, 1
Value Label:	0 = No
Value Label:	1 = Yes

Question 32:	How is the credit applied to tax liability?
Question Type:	Categorical - check all that apply
Variable Name:	EITC_SingOneChildCreditType_Dollar Amount
Variable Values:	0, 1
Value Label:	0 = No
Value Label:	1 = Yes
Question 33:	How is the credit applied to tax liability?
Question Type:	Categorical - check all that apply
Variable Name:	EITC_SingOneChildCreditType_Percentage
Variable Values:	0, 1
Value Label:	0 = No
Value Label:	1 = Yes
Question 34:	How is the credit applied to tax liability?
Question Type:	Categorical - check all that apply
Variable Name:	EITC_SingOneChildCreditType_Exempt from State Income Tax
Variable Values:	0, 1
Value Label:	0 = No
Value Label:	1 = Yes
Question 35:	How is the credit applied to tax liability?
Question Type:	Categorical - check all that apply
Variable Name:	EITC_SingOneChildCreditType_Credit type not specified in law
Variable Values:	0, 1
Value Label:	0 = No
Value Label:	1 = Yes
Question 36:	What is the dollar amount of the credit?
Question Type:	Currency Field
Variable Name:	EITC_SingOneChildDollAmt
Variable Values:	N/A
Question 37:	What is the EITC percentage for single individuals with one child?
Question Type:	Numeric Field
Variable Name:	EITC_SingOneChildPercent
Variable Values:	N/A
Question 38:	What is the EITC percentage applied to?
Question Type:	Categorical- Mutually Exclusive
Variable Name:	EITC_SingOneChild PercentApplied
Variable Values:	1, 2, 3, 4, 5, 6

Value Label:	1 = Federal EITC
Value Label:	2 = Total earned income
Value Label:	3 = EITC credit fund
Value Label:	4 = State income tax liability
Value Label:	5 = $12,000 minus individual's total income
Value Label:	6 = Not applicable
Question 39:	What is the EITC phase out percentage for single individuals with one child?
Question Type:	Numeric Field
Variable Name:	EITC_SingOneChildPhaseoutPercent
Variable Values:	N/A
Question 40:	What is the earned income amount?
Question Type:	Categorical - mutually exclusive
Variable Name:	EITC_SingOneChildAmount
Variable Values:	1, 2, 3, 4
Value Label:	1 = Adopts federal law
Value Label:	2 = Specified in state law
Value Label:	3 = Ten thousand dollars or less
Value Label:	4 = Earned income amount not specified in the law
Question 41:	What is the dollar amount of the credit?
Question Type:	Currency Field
Variable Name:	EITC_SingOneChildDollAmount
Variable Values:	N/A
Question 42:	What is threshold phase out AGI for single individuals with one child?
Question Type:	Categorical - mutually exclusive
Variable name:	eitc-singonechildthrphaseagi
Value Label:	1 = Adopts federal law
Value Label:	2 = Specified in state law
Value Label:	3 = Threshold phase out AGI not specified in the law
Question 43:	What is the applicable dollar amount?
Question Type:	Currency Field
Variable Name:	eitc-singonechildthrphaseagiamt
Variable Values:	N/A
Question 44:	What is the maximum EITC eligible AGI for single individuals with one child?
Question Type:	Categorical - mutually exclusive
Variable Name:	EITC_SingOneChildMaxAGI
Variable Values:	1, 2, 3

Value Label:	1 = Adopts federal law
Value Label:	2 = Specified in state law
Value Label:	3 = Maximum EITC eligible AGI not specified in the law
Question 45:	What is the applicable dollar amount?
Question Type:	Currency Field
Variable Name:	EITC_SingOneChildMaxAGIAmt
Variable Values:	N/A
Question 46:	Does the law provide an EITC for single individuals with two children?
Question Type:	Binary - mutually exclusive
Variable Name:	EITC_SingTwoChild
Variable Values:	0, 1
Value Label:	0 = No
Value Label:	1 = Yes
Question 47:	How is the credit applied to tax liability?
Question Type:	Categorical - check all that apply
Variable Name:	EITC_CreditTypeSingTwo_Dollar Amount
Variable Values:	0, 1
Value Label:	0 = No
Value Label:	1 = Yes
Question 48:	How is the credit applied to tax liability?
Question Type:	Categorical - check all that apply
Variable Name:	EITC_CreditTypeSingTwo_Percentage
Variable Values:	0, 1
Value Label:	0 = No
Value Label:	1 = Yes
Question 49:	How is the credit applied to tax liability?
Question Type:	Categorical - check all that apply
Variable Name:	EITC_CreditTypeSingTwo_Exempt from State Income Tax
Variable Values:	0, 1
Value Label:	0 = No
Value Label:	1 = Yes
Question 50:	How is the credit applied to tax liability?
Question Type:	Categorical - check all that apply
Variable Name:	EITC_CreditTypeSingTwo_Credit type not specified in law
Variable Values:	0, 1
Value Label:	0 = No

Value Label:	1 = Yes
Question 51:	What is the dollar amount of the credit?
Question Type:	Currency Field
Variable Name:	EITC_SingTwoChildDollAmt
Variable Values:	N/A
Question 52:	What is the EITC percentage for single individuals with two children?
Question Type:	Numeric Field
Variable Name:	EITC_SingTwoChildPercent
Variable Values:	N/A
Question 53:	What is the EITC percentage applied to?
Question Type:	Categorical- Mutually Exclusive
Variable Name:	EITC_SingTwoChild PercentApplied
Variable Values:	1, 2, 3, 4, 5, 6
Value Label:	1 = Federal EITC
Value Label:	2 = Total earned income
Value Label:	3 = EITC credit fund
Value Label:	4 = State income tax liability
Value Label:	5 = $12,000 minus individual's total income
Value Label:	6 = Not applicable
Question 54:	What is the EITC phase out percentage for single individuals with two children?
Question Type:	Numeric Field
Variable Name:	EITC_SingTwoChildPhaseoutPercent
Variable Values:	N/A
Question 55:	What is the earned income amount?
Question Type:	Categorical - mutually exclusive
Variable Name:	EITC_SingTwoChildAmount
Variable Values:	1, 2, 3, 4
Value Label:	1 = Adopts federal law
Value Label:	2 = Specified in state law
Value Label:	3 = Ten thousand dollars or less
Value Label:	4 = Earned income amount not specified in the law
Question 56:	What is the dollar amount of the credit?
Question Type:	Currency Field
Variable Name:	EITC_SingTwoChildDollAmount
Variable Values:	N/A
Question 57:	What is threshold phase out AGI for single individuals with two children?

Question Type:	Categorical - mutually exclusive
Variable name:	eitc-singtwochildthrphaseagi
Value Label:	1 = Adopts federal law
Value Label:	2 = Specified in state law
Value Label:	3 = Threshold phase out AGI not specified in the law
Question 58:	What is the applicable dollar amount?
Question Type:	Currency Field
Variable Name:	eitc-singtwochildthrphaseagiamt
Variable Values:	N/A
Question 59:	What is the maximum EITC eligible AGI for single individuals with two children?
Question Type:	Categorical - mutually exclusive
Variable Name:	EITC_SingTwoChildMaxAGI
Variable Values:	1, 2, 3
Value Label:	1 = Adopts federal law
Value Label:	2 = Specified in state law
Value Label:	3 = Maximum EITC eligible AGI not specified in the law
Question 60:	What is the applicable dollar amount?
Question Type:	Currency Field
Variable Name:	EITC_SingTwoChildMaxAGIAmt
Variable Values:	N/A
Question 61:	Does the law provide an EITC for single individuals with three or more children?
Question Type:	Binary - mutually exclusive
Variable Name:	EITC_SingThreeChild
Variable Values:	0, 1
Value Label:	0 = No
Value Label:	1 = Yes
Question 62:	How is the credit applied to tax liability?
Question Type:	Categorical - check all that apply
Variable Name:	EITC_CreditTypeSingThree_Dollar Amount
Variable Values:	0, 1
Value Label:	0 = No
Value Label:	1 = Yes
Question 63:	How is the credit applied to tax liability?
Question Type:	Categorical - check all that apply
Variable Name:	EITC_CreditTypeSingThree_Percentage
Variable Values:	0, 1

Value Label:	0 = No
Value Label:	1 = Yes
Question 64:	How is the credit applied to tax liability?
Question Type:	Categorical - check all that apply
Variable Name:	EITC_CreditTypeSingThree_Exempt from state Income Tax
Variable Values:	0, 1
Value Label:	0 = No
Value Label:	1 = Yes
Question 65:	How is the credit applied to tax liability?
Question Type:	Categorical - check all that apply
Variable Name:	EITC_CreditTypeSingThree_Credit type not specified in law
Variable Values:	0, 1
Value Label:	0 = No
Value Label:	1 = Yes
Question 66:	What is the dollar amount of the credit?
Question Type:	Currency Field
Variable Name:	EITC_SingThreeChildDollAmt
Variable Values:	N/A
Question 67:	What is the EITC percentage for single individuals with three or more children?
Question Type:	Numeric Field
Variable Name:	EITC_SingThreeChildPercent
Variable Values:	N/A
Question 68:	What is the EITC percentage applied to?
Question Type:	Categorical- Mutually Exclusive
Variable Name:	EITC_SingThreeChild PercentApplied
Variable Values:	1, 2, 3, 4, 5, 6
Value Label:	1 = Federal EITC
Value Label:	2 = Total earned income
Value Label:	3 = EITC credit fund
Value Label:	4 = State income tax liability
Value Label:	5 = $12,000 minus individual's total income
Value Label:	6 = Not applicable
Question 69:	What is the EITC phase out percentage for single individuals with three or more children?
Question Type:	Numeric Field
Variable Name:	EITC_SingThreeChildPhaseoutPercent
Variable Values:	N/A

Question 70:	What is the earned income amount?
Question Type:	Categorical - mutually exclusive
Variable Name:	EITC_SingThreeChildAmount
Variable Values:	1, 2, 3, 4
Value Label:	1 = Adopts federal law
Value Label:	2 = Specified in state law
Value Label:	3 = Ten thousand dollars or less
Value Label:	4 = Earned income amount not specified in the law
Question 71:	What is the dollar amount of the credit?
Question Type:	Currency Field
Variable Name:	EITC_SingThreeChildDollAmount
Variable Values:	N/A
Question 72:	What is threshold phase out AGI for single individuals with three children?
Question Type:	Categorical - mutually exclusive
Variable name:	eitc-singthreeechildthrphaseagi
Value Label:	1 = Adopts federal law
Value Label:	2 = Specified in state law
Value Label:	3 = Threshold phase out AGI not specified in the law
Question 73:	What is the applicable dollar amount?
Question Type:	Currency Field
Variable Name:	eitc-singthreechildthrphaseagiamt
Variable Values:	N/A
Question 74:	What is the maximum EITC eligible AGI for single individuals with three or more children?
Question Type:	Categorical - mutually exclusive
Variable Name:	EITC_SingThreeChildMaxAGI
Variable Values:	1, 2, 3
Value Label:	1 = Adopts federal law
Value Label:	2 = Specified in state law
Value Label:	3 = Maximum EITC eligible AGI not specified in the law
Question 75:	What is the applicable dollar amount?
Question Type:	Currency Field
Variable Name:	EITC_SingThreeChildMaxAGIAmt
Variable Values:	N/A
Question 76:	Does the law provide an EITC for married individuals with no children?
Question Type:	Binary - mutually exclusive
Variable Name:	EITC_MarNoChild

Variable Values:	0, 1
Value Label:	0 = No
Value Label:	1 = Yes
Question 77:	What is the eligible age range requirement for married individuals with no children?
Question Type:	Categorical - mutually exclusive
Variable Name:	EITC_MarNoChildAge
Variable Values:	1, 2, 3
Value Label:	1 = Adopts federal law
Value Label:	2 = Specified in state law
Value Label:	3 = Eligible age range requirement not specified in the law
Question 78:	What is the minimum age?
Question Type:	Numeric field
Variable Name:	EITC_MarNoChildMinAge
Variable Values:	N/A
Question 79:	What is the maximum age?
Question Type:	Numeric field
Variable Name:	EITC_MarNoChildMaxAge
Variable Values:	N/A
Question 80:	How is the credit applied to tax liability?
Question Type:	Categorical - check all that apply
Variable Name:	EITC_CreditTypeMarNo_Dollar amount
Variable Values:	0, 1
Value Label:	0 = No
Value Label:	1 = Yes
Question 81:	How is the credit applied to tax liability?
Question Type:	Categorical - check all that apply
Variable Name:	EITC_CreditTypeMarNo_Percentage
Variable Values:	0, 1
Value Label:	0 = No
Value Label:	1 = Yes
Question 82:	How is the credit applied to tax liability?
Question Type:	Categorical - check all that apply
Variable Name:	EITC_CreditTypeMarNo_Exempt from state Income Tax
Variable Values:	0, 1
Value Label:	0 = No
Value Label:	1 = Yes

Question 83:	How is the credit applied to tax liability?
Question Type:	Categorical - check all that apply
Variable Name:	EITC_CreditTypeMarNo_Credit type not specified in law
Variable Values:	0, 1
Value Label:	0 = No
Value Label:	1 = Yes
Question 84:	What is the dollar amount of the credit?
Question Type:	Currency Field
Variable Name:	EITC_MarNoChildDollAmt
Variable Values:	N/A
Question 85:	What is the EITC percentage for married individuals with no children?
Question Type:	Numeric Field
Variable Name:	EITC_MarNoChildPercent
Variable Values:	N/A
Question 86:	What is the EITC percentage applied to?
Question Type:	Categorical- Mutually Exclusive
Variable Name:	EITC_MarNoChild PercentApplied
Variable Values:	1, 2, 3, 4, 5, 6
Value Label:	1 = Federal EITC
Value Label:	2 = Total earned income
Value Label:	3 = EITC credit fund
Value Label:	4 = State income tax liability
Value Label:	5 = $12,000 minus individual’s total income
Value Label:	6 = Not applicable
Question 87:	What is the EITC phase out percentage for married individuals with no children?
Question Type:	Numeric Field
Variable Name:	EITC_MarNoChildPhaseoutPercent
Variable Values:	N/A
Question 88:	What is the earned income amount?
Question Type:	Categorical - mutually exclusive
Variable Name:	EITC_MarNoChildAmount
Variable Values:	1, 2, 3, 4
Value Label:	1 = Adopts federal law
Value Label:	2 = Specified in state law
Value Label:	3 = Ten thousand dollars or less
Value Label:	4 = Earned income amount not specified in the law

Question 89:	What is the dollar amount of the credit?
Question Type:	Currency Field
Variable Name:	EITC_MarNoChildDollAmount
Variable Values:	N/A
Question 90:	What is threshold phase out AGI for married individuals with no children?
Question Type:	Categorical - mutually exclusive
Variable name:	eitc-marnochildthrphaseagi
Value Label:	1 = Adopts federal law
Value Label:	2 = Specified in state law
Value Label:	3 = Threshold phase out AGI not specified in the law
Question 91:	What is the applicable dollar amount?
Question Type:	Currency Field
Variable Name:	eitc-marnochildthrphaseagiamt
Variable Values:	N/A
Question 92:	What is the maximum EITC eligible AGI for married individuals with no children?
Question Type:	Categorical - mutually exclusive
Variable Name:	EITC_MarNoChildMaxAGI
Variable Values:	1, 2, 3
Value Label:	1 = Adopts federal law
Value Label:	2 = Specified in state law
Value Label:	3 = Maximum EITC eligible AGI not specified in the law
Question 93:	What is the applicable dollar amount?
Question Type:	Currency Field
Variable Name:	EITC_MarNoChildMaxAGIAmt
Variable Values:	N/A
Question 94:	Does the law provide an EITC for married individuals with one child?
Question Type:	Binary - mutually exclusive
Variable Name:	EITC_MarOneChild
Variable Values:	0, 1
Value Label:	0 = No
Value Label:	1 = Yes
Question 95:	How is the credit applied to tax liability?
Question Type:	Categorical - check all that apply
Variable Name:	EITC_CreditTypeMarOne_Dollar Amount
Variable Values:	0, 1
Value Label:	0 = No

Value Label:	1 = Yes
Question 96:	How is the credit applied to tax liability?
Question Type:	Categorical - check all that apply
Variable Name:	EITC_CreditTypeMarOne_Percentage
Variable Values:	0, 1
Value Label:	0 = No
Value Label:	1 = Yes
Question 97:	How is the credit applied to tax liability?
Question Type:	Categorical - check all that apply
Variable Name:	EITC_CreditTypeMarOne_Exempt from state Income Tax
Variable Values:	0, 1
Value Label:	0 = No
Value Label:	1 = Yes
Question 98:	How is the credit applied to tax liability?
Question Type:	Categorical - check all that apply
Variable Name:	EITC_CreditTypeMarOne_Credit type not specified in law
Variable Values:	0, 1
Value Label:	0 = No
Value Label:	1 = Yes
Question 99:	What is the dollar amount of the credit?
Question Type:	Currency Field
Variable Name:	EITC_MarOneChildDollAmt
Variable Values:	N/A
Question 100:	What is the EITC percentage for married individuals with one child?
Question Type:	Numeric Field
Variable Name:	EITC_MarOneChildPercent
Variable Values:	N/A
Question 101:	What is the EITC percentage applied to?
Question Type:	Categorical- Mutually Exclusive
Variable Name:	EITC_MarOneChild PercentApplied
Variable Values:	1, 2, 3, 4, 5, 6
Value Label:	1 = Federal EITC
Value Label:	2 = Total earned income
Value Label:	3 = EITC credit fund
Value Label:	4 = State income tax liability
Value Label:	5 = $12,000 minus individual’s total income

Value Label:	6 = Not applicable
Question 102:	What is the EITC phase out percentage for married individuals with one child?
Question Type:	Numeric Field
Variable Name:	EITC_MarOneChildPhaseoutPercent
Variable Values:	N/A
Question 103:	What is the earned income amount?
Question Type:	Categorical - mutually exclusive
Variable Name:	EITC_MarOneChildAmount
Variable Values:	1, 2, 3, 4
Value Label:	1 = Adopts federal law
Value Label:	2 = Specified in state law
Value Label:	3 = Ten thousand dollars or less
Value Label:	4 = Earned income amount not specified in the law
Question 104:	What is the dollar amount of the credit?
Question Type:	Currency Field
Variable Name:	EITC_MarOneChildDollAmount
Variable Values:	N/A
Question 105:	What is threshold phase out AGI for married individuals with one child?
Question Type:	Categorical - mutually exclusive
Variable name:	eitc-maronechildthrphaseagi
Value Label:	1 = Adopts federal law
Value Label:	2 = Specified in state law
Value Label:	3 = Threshold phase out AGI not specified in the law
Question 106:	What is the applicable dollar amount?
Question Type:	Currency Field
Variable Name:	eitc-maronechildthrphaseagiamt
Variable Values:	N/A
Question 107:	What is the maximum EITC eligible AGI for married individuals with one child?
Question Type:	Categorical - mutually exclusive
Variable Name:	EITC_MarOneChildMaxAGI
Variable Values:	1, 2, 3
Value Label:	1 = Adopts federal law
Value Label:	2 = Specified in state law
Value Label:	3 = Maximum EITC eligible AGI not specified in the law
Question 108:	What is the applicable dollar amount?
Question Type:	Currency field

Variable Name:	EITC_MarOneChildMaxAGIAmt
Variable Values:	N/A
Question 109:	Does the law provide an EITC for married individuals with two children?
Question Type:	Binary - mutually exclusive
Variable Name:	EITC_MarTwoChild
Variable Values:	0, 1
Value Label:	0 = No
Value Label:	1 = Yes
Question 110:	How is the credit applied to tax liability?
Question Type:	Categorical - check all that apply
Variable Name:	EITC_CreditTypeMarTwo_Dollar amount
Variable Values:	0, 1
Value Label:	0 = No
Value Label:	1 = Yes
Question 111:	How is the credit applied to tax liability?
Question Type:	Categorical - check all that apply
Variable Name:	EITC_CreditTypeMarTwo_Percentage
Variable Values:	0, 1
Value Label:	0 = No
Value Label:	1 = Yes
Question 112:	How is the credit applied to tax liability?
Question Type:	Categorical - check all that apply
Variable Name:	EITC_CreditTypeMarTwo_Exempt from state Income Tax
Variable Values:	0, 1
Value Label:	0 = No
Value Label:	1 = Yes
Question 113:	How is the credit applied to tax liability?
Question Type:	Categorical - check all that apply
Variable Name:	EITC_CreditTypeMarTwo_Credit type not specified in law
Variable Values:	0, 1
Value Label:	0 = No
Value Label:	1 = Yes
Question 114:	What is the dollar amount of the credit?
Question Type:	Currency Field
Variable Name:	EITC_MarTwoChildDollAmt
Variable Values:	N/A

Question 115:	What is the EITC percentage for married individuals with two children?
Question Type:	Numeric Field
Variable Name:	EITC_MarTwoChildPercent
Variable Values:	N/A
Question 116:	What is the EITC percentage applied to?
Question Type:	Categorical- Mutually Exclusive
Variable Name:	EITC_MarTwoChild PercentApplied
Variable Values:	1, 2, 3, 4, 5, 6
Value Label:	1 = Federal EITC
Value Label:	2 = Total earned income
Value Label:	3 = EITC credit fund
Value Label:	4 = State income tax liability
Value Label:	5 = $12,000 minus individual's total income
Value Label:	6 = Not applicable
Question 117:	What is the EITC phase out percentage for married individuals with two children?
Question Type:	Numeric Field
Variable Name:	EITC_MarTwoChildPhaseoutPercent
Variable Values:	N/A
Question 118:	What is the earned income amount?
Question Type:	Categorical - mutually exclusive
Variable Name:	EITC_MarTwoChildAmount
Variable Values:	1, 2, 3, 4
Value Label:	1 = Adopts federal law
Value Label:	2 = Specified in state law
Value Label:	3 = Ten thousand dollars or less
Value Label:	4 = Earned income amount not specified in the law
Question 119:	What is the dollar amount of the credit?
Question Type:	Currency Field
Variable Name:	EITC_MarTwoChildDollAmount
Variable Values:	N/A
Question 120:	What is threshold phase out AGI for married individuals with two children?
Question Type:	Categorical - mutually exclusive
Variable name:	eitc-martwochildthrphaseagi
Value Label:	1 = Adopts federal law
Value Label:	2 = Specified in state law
Value Label:	3 = Threshold phase out AGI not specified in the law

Question 121:	What is the applicable dollar amount?
Question Type:	Currency Field
Variable Name:	eitc-martwochildthrphaseagiamt
Variable Values:	N/A
Question 122:	What is the maximum EITC eligible AGI for married individuals with two children?
Question Type:	Categorical - mutually exclusive
Variable Name:	EITC_MarTwoChildMaxAGI
Variable Values:	1, 2, 3
Value Label:	1 = Adopts federal law
Value Label:	2 = Specified in state law
Value Label:	3 = Maximum EITC eligible AGI not specified in the law
Question 123:	What is the applicable dollar amount?
Question Type:	Currency Field
Variable Name:	EITC_MarTwoChildMaxAGIAmt
Variable Values:	N/A
Question 124:	Does the law provide an EITC for married individuals with three or more children?
Question Type:	Binary - mutually exclusive
Variable Name:	EITC_MarThreeChild
Variable Values:	0, 1
Value Label:	0 = No
Value Label:	1 = Yes
Question 125:	How is the credit applied to tax liability?
Question Type:	Categorical - check all that apply
Variable Name:	EITC_CreditTypeMarThree_Dollar Amount
Variable Values:	0, 1
Value Label:	0 = No
Value Label:	1 = Yes
Question 126:	How is the credit applied to tax liability?
Question Type:	Categorical - check all that apply
Variable Name:	EITC_CreditTypeMarThree_Percentage
Variable Values:	0, 1
Value Label:	0 = No
Value Label:	1 = Yes
Question 127:	How is the credit applied to tax liability?
Question Type:	Categorical - check all that apply
Variable Name:	EITC_CreditTypeMarThree_Exempt from state Income Tax

Variable Values:	0, 1
Value Label:	0 = No
Value Label:	1 = Yes
Question 128:	How is the credit applied to tax liability?
Question Type:	Categorical - check all that apply
Variable Name:	EITC_CreditTypeMarThree_Credit type not specified in law
Variable Values:	0, 1
Value Label:	0 = No
Value Label:	1 = Yes
Question 129:	What is the dollar amount of the credit?
Question Type:	Currency Field
Variable Name:	EITC_MarThreeChildDollAmt
Variable Values:	N/A
Question 130:	What is the EITC percentage for married individuals with three or more children?
Question Type:	Numeric Field
Variable Name:	EITC_MarThreeChildPercent
Variable Values:	N/A
Question 131:	What is the EITC percentage applied to?
Question Type:	Categorical- Mutually Exclusive
Variable Name:	EITC_MarThreeChild PercentApplied
Variable Values:	1, 2, 3, 4, 5, 6
Value Label:	1 = Federal EITC
Value Label:	2 = Total earned income
Value Label:	3 = EITC credit fund
Value Label:	4 = State income tax liability
Value Label:	5 = $12,000 minus individual’s total income
Value Label:	6 = Not applicable
Question 132:	What is threshold phase out AGI for married individuals with three children?
Question Type:	Categorical - mutually exclusive
Variable name:	eitc-marthreeechildthrphaseagi
Value Label:	1 = Adopts federal law
Value Label:	2 = Specified in state law
Value Label:	3 = Threshold phase out AGI not specified in the law
Question 133:	What is the applicable dollar amount?
Question Type:	Currency Field
Variable Name:	eitc-marthreechildthrphaseagiamt

Variable Values:	N/A
Question 134:	What is the earned income amount?
Question Type:	Categorical - mutually exclusive
Variable Name:	EITC_MarThreeChildAmount
Variable Values:	1, 2, 3, 4
Value Label:	1 = Adopts federal law
Value Label:	2 = Specified in state law
Value Label:	3 = Ten thousand dollars or less
Value Label:	4 = Earned income amount not specified in the law
Question 135:	What is the dollar amount of the credit?
Question Type:	Currency Field
Variable Name:	EITC_MarThreeChildDollAmount
Variable Values:	N/A
Question 136:	What is the threshold phase out AGI for married individuals with three or more children?
Question Type:	Currency Field
Variable Name:	EITC_MarThreeChild ThreshPhaseAGI
Variable Values:	N/A
Question 137:	What is the maximum EITC eligible AGI for married individuals with three or more children?
Question Type:	Categorical - mutually exclusive
Variable Name:	EITC_MarThreeChildMaxAGI
Variable Values:	1, 2, 3
Value Label:	1 = Adopts federal law
Value Label:	2 = Specified in state law
Value Label:	3 = Maximum EITC eligible AGI not specified in the law
Question 138:	What is the applicable dollar amount?
Question Type:	Currency Field
Variable Name:	EITC_MarThreeChildMaxAGIAmt
Variable Values:	N/A
