# Supplementary material for: Anti-poverty policy and health: Attributes and diffusion of state earned income tax credits across U.S. states from 1980 to 2020
Source: PLoS One. 2020 Nov 20;15(11):e0242514. doi: 10.1371/journal.pone.0242514 (PMC7678980; doi:10.1371/journal.pone.0242514)
Supplement: S2 Appendix — (DOCX) [file pone.0242514.s002.docx]

Research Protocol for Earned Income Tax Credit

Prepared by the Policy Surveillance Program Staff

July 2016

Earned Income Tax Credit

July 2016

Research Protocol

# Date of Protocol: July 15, 2013; September 24, 2015; February 1, 2016, July 1, 2016

1. **Scope:** Compile state and federal statutes and regulations regarding eligibility and benefits under federal, state, and local earned income tax credit (EITC) laws. The scope of the dataset includes any states that enact tax credits for earned income as well as the federal EITC law. This longitudinal dataset includes coding questions about eligibility for the credit, the governing authority for eligibility, credit phase outs, and refunding excess credit over tax liability.
2. **Project team:** Nicolas Wilhelm, JD; Sarah Klieger, MPH; Andrew Campbell, JD; Adrienne Ghorashi, JD; Andrew Kunka, JD;
3. **Primary Data Collection**
   1. **Project dates**: June 15, 2013 – August 15, 2013
   2. **Dates covered in the dataset**: This dataset is longitudinal, and spans the period of time between January 1, 1980 and July 1, 2013. There have since been updates to the dataset, bringing it forward in time to February 1, 2016 (see sections below for more information on updates).
   3. **Data Collection Methods**: Discuss scoping steps (background memo, 5 state memo). How the sources were collected.
      1. **Databases used:**

Searches were conducted using WestlawNext and state-specific legislature websites. Full text versions of the laws were collected from state legislature websites.

- - 1. **Search terms used:**
       1. Adv: “earned income tax credit” % housing
       2. Adv: “low income tax credit” % housing
       3. Adv: “Working Families” /p “Tax Credit”
       4. Adv: “Family Tax Credit”
       5. Adv: “Refundable Tax Credit”
       6. The Researchers supplemented key word searches by examination of the table of contents of the tax section of each state code. Search results were compared with secondary sources to confirm that all relevant law was being collected by the Researchers.
    2. **Initial Returns and Additional Inclusion or Exclusion Criteria:**

Statutes and regulations were included if they outlined eligibility or benefit information for tax credits aimed at providing tax relief or refunds to low income working taxpayers. Statutes providing credits to low income workers for assistance with food, housing, or child care were not included.

Once relevant statutes and regulations were identified, a master sheet was created outlining all the relevant statutes and administrative materials for that jurisdiction as well as the legislative history for those laws. Session laws were collected for each amendment beginning in January 1, 1980. Using the statutory history and session laws, full text versions of the law were created to cover the entire relevant time period. The law was then entered into the LawAtlas Workbench for coding.

1. **Coding**
   1. **Development of coding questions:** To develop coding questions, the Researchers and Supervisor met as a group to narrow the scope of the dataset from all topics related to low income tax credits to specific topics that would be of interest to the dataset users. The group conceptualized coding questions to properly gather information on those specific topics. The Researchers drafted the conceptualized questions, and then circulated them for review by the Supervisor until the group was satisfied with them. Once the coding questions were finalized, Researcher #1 entered the questions into the LawAtlas Workbench.
   2. **Dataset terminology:**
      1. “Earned Income Tax Credit” (EITC) is a credit for low or moderate income taxpayers who meet state or federal eligibility requirements, including the definition of “earned income,” which is typically work-related income.
      2. “Low Income Tax Credit” (LITC) is a state credit or refund for low or moderate income taxpayers that includes all income to determine eligibility regardless of whether the income was earned income.
      3. “Earned income amount” is the work-related, taxable income amount that is used to determine eligibility for the EITC.
      4. “Adjusted Gross Income (AGI) is an individual’s gross income minus adjustments (or deductions).
      5. “Threshold phaseout AGI” is the AGI at which the EITC begins to decrease by a set percentage until the EITC reaches zero.
      6. Phaseout percentage” is the rate (calculated as set a percentage of income) at which an individual or household’s EITC begins to decrease from the threshold phaseout AGI until the EITC reaches zero.
   3. **Coding rules:** Researcher #1 and Researcher #2 were responsible for coding the dataset in the Workbench. The Researchers were assigned a number of states to code independently.
      1. The law was coded as it applies to full-time state residents.
      2. If the law creates a distinction between unmarried taxpayers filing as “single” or as “head of household,” single was coded for unmarried taxpayers with no children and head of household was coded for unmarried taxpayers with one or more child.
      3. If a state adopts federal standards for state earned income eligibility, questions regarding eligibility were coded “adopts federal law.”
      4. For example, for the question: “What is the maximum EITC eligible AGI for X?”: Researchers coded the question with “adopts federal law” when it was clear the state had adopted federal eligibility requirements.
      5. If a question did not apply for the law in that jurisdiction, the question was coded “N/A”.
      6. When a state lists a credit percentage and references the federal law with a different percentage, coders used the state’s percentage in coding.
      7. The effective date used in the dataset is January 1 of the applicable tax year or taxable year for that iteration if indicated explicitly in law, or the effective date of the most recent law if no applicable tax year is listed.
2. **Quality Control**
   1. **Quality Control – Coding:**
      1. **Redundant coding:** Twenty percent of the records were redundantly coded by both Researcher #1 and Researcher #2. This means two records were created containing the same law, questions and date. Both Researcher #1 and Researcher #2 coded these records, resulting in two records for the same jurisdiction and date. Records were selected for redundant coding using the random number generator in Microsoft Excel.

The Supervisor reviewed the redundant coding by downloading the data from the Workbench into Microsoft Excel and comparing the records, variable by variable, looking for divergences. When a divergence was identified it was discussed with the researchers. The reason for the divergence was identified and resolved. A measure of divergence was calculated by the Supervisor and the duplicate record was deleted. The overall rate of divergence for the duplicate coding was 5%.

- - 1. **Post-production quality control:** The Supervisor oversaw the overall quality of the data by downloading the data from the Workbench in Microsoft Excel and reviewing it in order to find caution flags, divergences in redundant records and errors in the coding. Daily Coding Review sheets were sent to the researchers for their review. Issues in the coding were discussed by the Supervisor and the researchers in coding meetings.

Once Researcher #1 and Researcher #2 completed coding, a third researcher (“Researcher #3”), who was completely naïve to the project was brought in to duplicate code an additional twenty percent of the records. Researcher #3 was assigned records using the random number generator in Microsoft Excel.

Once Researcher #3 completed coding the Supervisor downloaded the data into Microsoft excel. The data was once again reviewed, variable by variable, looking for divergences in the coding. When a divergence was identified, it was discussed with the researchers. The reason for the divergence was identified and resolved. A measure of divergence was calculated by the Supervisor and the duplicate record was deleted. The overall rate of divergence for the naïve coder was 7%.

1. **Update: September 2015**
   1. **Scope:** For the September 2015 update, the team tracked EITC laws between July 1, 2013 and September 1, 2015.
   2. **Data Collection:** The Researchers went through the coded laws in every jurisdiction looking for any amendments. The Researchers also conducted general searches using the search terms in this protocol to check for any newly-enacted laws. Finally, the Researchers checked the latest entry in each state for possible updates within the legal text (E.g., future applicable tax years listed within the legal text itself, as opposed to the credit history). The Supervisor checked their findings, and looked for any new EITC laws within our scope. The team found that 10 states required updates: Colorado, Maryland, Oregon, Rhode Island, Connecticut, Indiana, Iowa, Maine, Nebraska, and New Jersey. The Researchers created new records with updated legal text for states with new laws or changes to existing laws that affected answer choices.
   3. **Coding Updated Findings:**
      1. **Coding methods**: The Researchers and the Supervisor located and collected relevant statutes in accordance with the inclusion/exclusion determinations described in subsection entitled **Primary Data Collection**, above. The team found that 10 states: Colorado, Maryland, Oregon, Rhode Island, Connecticut, Indiana, Iowa, Maine, Nebraska, and New Jersey had updates to existing laws that required coding. The Researchers also found that California had a newly-enacted EITC law, which was collected and coded.
         1. The Researchers excerpted the legal text if the applicable statute(s) included tax credits besides EITC. For excerpts, the effective date is updated and a new iteration is added only when there is a change to the included legal text, not the excerpted portions..
         2. A valid-through date of September 1, 2015 was selected because the Researchers updated records through that date.
         3. For the question, “What is the maximum EITC eligible AGI for X?” When Researchers answer “adopts federal law,” there are no citations.
   4. **Quality Control:**
      1. After the Researchers coded the new entries, the Supervisor performed quality control. The Supervisor downloaded all coding data into Microsoft Excel and examined the data for any missing answers, incorrect citations, and caution notes.
      2. The Researchers redundantly coded 20% of the newly-added records. The Supervisor downloaded the redundantly coded records in Excel and identified coding errors and divergences between originally and redundantly coded records. There were 2 substantive divergences; the rate of divergence was under 10%. The divergences and caution notes were discussed and resolved by the Supervisor and Researchers in a coding review meeting.
2. **Update: February 2016**
   1. **Scope:** For the February 2016 update, we have been tracking EITC laws from September 1, 2015 to February 1, 2016.
   2. **Data Collection:** The Researchers went through the coded laws in every jurisdiction looking for any amendments. The Researchers also conducted general searches using the search terms in this protocol to check for any newly-enacted laws. Finally, the Researchers checked the latest entry in each state for possible updates within the legal text (E.g., future applicable tax years listed within the legal text itself, as opposed to the credit history). The Supervisor checked their findings, and looked for any new EITC laws within our scope. The team found that 7 states required updates: the District of Columbia, Maine, Maryland, Massachusetts, Michigan, Ohio, and Pennsylvania. The Researchers created new records with updated legal text for states with new laws or changes to existing laws that affected answer choices.
   3. **Coding Updated Findings:**
      1. **Coding methods**: The Researchers and the Supervisor located and collected relevant statutes in accordance with the inclusion/exclusion determinations described in subsection entitled **Primary Data Collection**, above. The team found that 7 states: the District of Columbia, Maine, Maryland, Massachusetts, Michigan, Ohio, and Pennsylvania had updates to existing laws that required coding.
         1. The Researchers excerpted the legal text if the applicable statute(s) included tax credits besides EITC. For excerpts, the effective date is updated and a new iteration is added only when there is a change to the included legal text, not the excerpted portions.
         2. A valid-through date of February 1, 2016 was selected because the Researchers updated records through that date.
         3. For the question, “What is the maximum EITC eligible AGI for X?” When Researchers answer “adopts federal law,” there are no citations.
   4. **Quality Control:**
      1. After the Researchers coded the new entries, the Supervisor performed quality control. The Supervisor downloaded all coding data into Microsoft Excel and examined the data for any missing answers, incorrect citations, and caution notes.
      2. The Researchers redundantly coded 20% of the newly-added records. The Supervisor downloaded the redundantly coded records in Excel and identified coding errors and divergences between originally and redundantly coded records. There were 58 substantive divergences out of 600 potential divergences; the rate of divergence was 9.667%. The divergences and caution notes were discussed and resolved by the Supervisor and Researchers in a coding review meeting.
3. **Update: July 2016**
   1. **Scope:** For the July 2016 update, the team converted the following questions from text based questions to multiple choice questions, in order to increase consistency in how responses were captured:
      1. What is the required age for ____?
      2. What is the earned income amount?
      3. What is the threshold phase out AGI for ____?
      4. What is the maximum EITC eligible AGI for_____?
   2. **Quality Control:** Quality control was performed on a sample of five states as the dataset was recoded. Two researchers independently coded all records for these five jurisdictions. Their responses were compared by the Supervisor. Out of 10960 potential responses, there were 484 divergences, for a divergence rate of 4.42%. All divergences were resolved, and other jurisdictions were then checked and resolved for similar divergences.
   3. **Post-production quality control:** The Supervisor oversaw the overall quality of the data by downloading the data from the Workbench in Microsoft Excel and reviewing it in order to find caution flags, divergences in redundant records and errors in the coding. When data collection was finalized, our statistics expert paneled the data by month, checked for date gaps, and looked for outliers which were then checked for errors.
