# Supplementary material for: Anti-poverty policy and health: Attributes and diffusion of state earned income tax credits across U.S. states from 1980 to 2020
Source: PLoS One. 2020 Nov 20;15(11):e0242514. doi: 10.1371/journal.pone.0242514 (PMC7678980; doi:10.1371/journal.pone.0242514)
Supplement: S3 Appendix — (DOCX) [file pone.0242514.s003.docx]

Supplemental 3 File: EITC Data Codebook 2020

State: State Abbreviation

Year: Applicable tax year (1980 through 2020)

FIPS: State FIPS Code

State_income_tax:

0- no state income tax

1- state has an income tax

Perc_0: The percent of the federal credit for households with no children

Perc_1: The percent of the federal credit for households with 1 child

Perc_2: The percent of the federal credit for households with 2 children

Perc_3: The percent of the federal credit for households with 3 or more children

Refundable:

MISSING - State has no EITC, so refundability not applicable

0- State has an EITC that is not refundable

1- State has an EITC that is refundable

Notes:

-For a minority of states that do not base their EITC on the federal EITC,

percentages were calculated and not taken directly from legal text.

See Komro et al 2020, table 1 for details

-VA in 2000 to 2005 is intentionally left missing for Perc_0-Perc_3. During

those years, VA had a fixed credit based on the number of persons in a

household that applied to households under the federal poverty guidelines.

Users are encouraged to modify these rows as needed for their specific

analyses.

- Maryland appears twice in the data set as MD1 and MD2. Maryland has both a

refundable and non-refundable EITC credit that were enacted at different points.

For years when both are available, residents must choose one or the other. Both

are provided in the data so that users can chose which is more relevant for

their specific analysis.

- Washington enacted a state EITC in 2009. However, the credit has never been

funded. Accordingly, for analytic purposes we treat WA as having no credit

- The Colorado EITC was established as a refund mechanism under CO’s Taxpayer

Bill of Rights (TABOR), and requires a budget surplus for the EITC credit to

be financed. The credit was funded from 1999-2001. No surplus was available

from 2002 to 2014. The credit was again funded in 2015. From 2016 on, a permanent

EITC was implemented. Years without funding are treated as having no EITC.

- Beginning in 2017, Oregon enacted an additional child tax credit worth 3% of the federal EITC.

To qualify for this credit, you must qualify for the federal EITC as well as have a qualifying

dependent under 3 years of age. This additional child tax credit is not included in the data.

Analysts wishing to modify this for analyses of qualifying samples should add 3% to the relevant

years in Oregon.
