# Supplementary material for: Anti-poverty policy and health: Attributes and diffusion of state earned income tax credits across U.S. states from 1980 to 2020
Source: PLoS One. 2020 Nov 20;15(11):e0242514. doi: 10.1371/journal.pone.0242514 (PMC7678980; doi:10.1371/journal.pone.0242514)
Supplement: S5 Appendix — (DOCX) [file pone.0242514.s005.docx]

**Research Protocol for State Earned Income Tax Credit Laws**

Prepared by the Office of the Associate Director for Policy and Strategy, Office of the Director, Centers for Disease Control and Prevention

April 2020

**Research Protocol**

1. **Date of Protocol:** April 22, 2020
2. **Scope:** Updated select variables of an existing dataset of federal and state statutes and regulations that comprise earned income tax credit (EITC) laws. The original dataset was previously compiled by the Policy Surveillance Program within Temple University’s Public Health Law Research Program (Temple).

The 2020 update included a systematic collection, textual legal analysis, and numerical coding of all legislative changes to state EITC laws that effect the select variables from January 1, 2017 through March 1, 2020, in the 50 states and Washington, D.C. (collectively, states). During that process, some anomalies were identified in data that had been included in the original dataset, and the 2020 update expanded to include a retrospective quality control review and correction, as needed, of previously coded data. The 2020 update coded for select variables including amount of the state credit as a percent of the federal EITC; the amount of the state EITC stratified by number of children, if applicable; refundability of the state’s EITC; and the date upon which the legislative change took effect (i.e., the date after which an eligible taxpayer could claim the modified).

1. **Project Team**: Megan A. Kelly, JD, MPH; Dawn Pepin, JD, MPH
2. **Primary Data Collection**
   1. **Project dates**: January 10, 2020 – April 22, 2020
   2. **Dates covered in the dataset**: This updated longitudinal dataset spans the period from January 1, 1980, through March 1, 2020. The original dataset contained data through the end of 2016, and the 2020 update brought it current as of March 1, 2020.
   3. **Data collection methods**: Conducted independent review of literature to assess whether search terms reflect the current state of the laws; conducted sample searches for ten states to assess scope of search terms (i.e., sufficiently broad to capture all state EITC laws without returning a large number of unrelated laws); and expanded search terms based on review of the literature and results of sample searches to capture the laws in states known to have an EITC. After the search string was finalized, it was used to search for states with EITC laws.

The statutory history available in the WestlawNext database was searched for the effective date of the EITC for each legislative change, and if unavailable, the researchers consulted the respective session laws from the state’s legislative website.

- - 1. **Databases used**: Searches were conducted using WestlawNext and state-specific legislative websites.
    2. **Search terms used**: The initial search string was constructed from the search terms used by Temple to identify EITC laws. The search terms were expanded to reflect the current state of the laws based upon an independent review of the literature and sample searches of states known to have an EITC law. The search string evolved, and the final search string developed was as follows:
       1. adv: SD(("earned income tax credit" % housing) or ("earned income credit" % housing) or ("earned income" /5 (tax or credit) or ("low income tax credit" % housing)) or ("Working Families" /p "Tax Credit") or ("Family Tax Credit") or ("Refundable Tax Credit") or (“section 32” /s “Internal Revenue Code”))
    3. **Initial Returns and Additional Inclusion or Exclusion Criteria:**

State statutes were included if they identified eligibility criteria or benefit information for a tax credit applied against earned income for low-income working individuals (i.e., the credit did not have to be labelled as an Earned Income Tax Credit). Statutes that provided tax credits for assistance with food, housing, or childcare were not included, nor were credits applied against income that was not earned (i.e., investment income).

The effective date of the credit for each legislative change (i.e., date the modified credit became available) was obtained from a review of the statutory history, if available in the WestlawNext database, or the respective session law obtained from the state’s legislative website.

1. **Coding**
   1. **Development of coding questions:** The Researchers met with the colleagues from Emory University who had been involved in the creation of the original dataset. Together, they identified a subset of the previously developed questions to be coded during the update (i.e. the select variables of interest). After coding several sample states, the Researchers conferred with the Emory colleagues to clarify and specify the coding question related to the effective date of the EITC. A modified codebook was created to capture the questions coded during the 2020 update.
   2. **Coding rules:** Researcher 1 and Research 2 each coded all states independently. They applied the following rules to their coding:
      1. The law was coded as it applies to full-time state residents.
      2. If the law creates a distinction between unmarried taxpayers filing as “single” or as “head of household,” single was coded for unmarried taxpayers with no children and head of household was coded for unmarried taxpayers with one or more child.
      3. If a state did not adopt a federal eligibility criterion, a notation was made as to how the state eligibility criteria varied along with a comment about the impact on how the state EITC is operationalized.
      4. If a question did not apply for the law in that jurisdiction, the question was coded “N/A”.
      5. When a state did not represent its EITC as a percentage of the Federal EITC, an effective rate was calculated for the state, translating its credit to a percentage of the Federal EITC based on relative maximum credit amount and the income phaseout amount. A notation was made to indicate which states are coded with an effective rate, including a comment with the state’s actual EITC amount.
      6. The effective data represents the date on which the credit became available to claim for a given tax year, regardless of the effective date of the law providing for the credit. For example, a law that is effective as of May 15, 2020, may provide for an EITC that takes effect retrospectively, on January 1, 2020, or prospectively, “no earlier than December 31, 2020,” resulting in coding the credit effective date as January 1, 2020, and January 1, 2021, respectively.
2. **Quality Control**
   - 1. **Redundant coding**: All laws were coded redundantly by both Researcher 1 and Researcher 2. This means that both Researchers searched for, analyzed, and coded the laws in all 50 states. The Researchers compared the redundant coding for the 10 sample states and found de minimus divergence, which was resolved through discussion and consensus. The overall divergence rate for the sample state coding was 2%.
     2. **Post-production quality control**: After all coding was completed by both Researchers, they reviewed and compared their data for each variable in every state. When a diverge was identified, the Researchers discussed it to identify and resolve the reason for it. The overall rate of divergence for the completed coding was < 1%.
